# BC200 overexpression contributes to luminal and triple negative breast cancer pathogenesis

**DOI:** 10.1186/s12885-019-6179-y

**Published:** 2019-10-23

**Authors:** Maria Barton, Julia Santucci-Pereira, Olivia G. Vaccaro, Theresa Nguyen, Yanrong Su, Jose Russo

**Affiliations:** 1 Biochemistry Department, Lewis Katz School of Medicine, Temple University, Philadelphia, PA 19140 USA; 2 The Irma H. Russo, MD Breast Cancer Research Laboratory, Fox Chase Cancer Center, Temple University Health System, Philadelphia, PA 19111 USA

**Keywords:** Long non-coding RNAs, Breast cancer, TNBC, Luminal, Overexpression, Parity, Prevention

## Abstract

**Background:**

Long non coding RNAs (lncRNAs) are RNA molecules longer than 200 nucleotides that are not translated into proteins, but regulate the transcription of genes involved in different cellular processes, including cancer. Epidemiological analyses have demonstrated that parous women have a decreased risk of developing breast cancer in postmenopausal years if they went through a full term pregnancy in their early twenties. We here provide evidence of the role of BC200 in breast cancer and, potentially, in pregnancy’s preventive effect in reducing the lifetime risk of developing breast cancer.

**Methods:**

Transcriptome analysis of normal breast of parous and nulliparous postmenopausal women revealed that several lncRNAs are differentially expressed in the parous breast. RNA sequencing of healthy postmenopausal breast tissue biopsies from eight parous and eight nulliparous women showed that there are 42 novel lncRNAs differentially expressed between these two groups. Screening of several of these 42 lncRNAs by RT-qPCR in different breast cancer cell lines, provided evidence that one in particular, lncEPCAM (more commonly known as BC200), was a strong candidate involved in cancer progression. Proliferation, migration, invasion and xerograph studies confirmed this hypothesis.

**Results:**

The poorly studied oncogenic BC200 was selected to be tested in vitro and in vivo to determine its relevance in breast cancer and also to provide us with an understanding of its role in the increased susceptibility of the nulliparous women to cancer. Our results show that BC200 is upregulated in nulliparous women, and breast cancer cells and tissue. The role of BC200 is not completely understood in any of the breast cancer subtypes. We here provide evidence that BC200 has a role in luminal breast cancer as well as in the triple negative breast cancer subtype.

**Conclusion:**

When overexpressed in luminal and triple negative breast cancer cell lines, BC200 shows increased proliferation, migration, and invasion in vitro. In vivo, overexpression of BC200 increased tumor size. Although treatment for cancer using lncRNAs as targets is in its infancy, the advancement in knowledge and technology to study their relevance in disease could lead to the development of novel treatment and preventive strategies for breast cancer.

## Background

Breast cancer affects women of all ages, races and nationalities [1–3]. The worldwide incidence has increased 30% since the 1970s, well above lung & bronchus, colorectum, and uterine corpus [2]. In the USA only, it is estimated that at least 246,000 new cases of female breast cancer will be diagnosed each year, making breast cancer the second leading cause of cancer since 1990 [2]. Although often referred to as a single disease, breast cancer is distinguished by several distinct histologic subtypes and at least 4 different molecular subtypes (Luminal A, Luminal B, HER2+ and Triple Negative Breast Cancer [TNBC]). These 4 subtypes are associated with distinct risk factors and are biologically variable in presentation, development, and outcomes after treatment [4–6]. Overall, 74% of breast cancer cases are luminal type A, 12% are TNBC, 10% are luminal B, and 4% are HER2+ (HER2-enriched), with the distributions varying by race and ethnicity as reported by the American Cancer Society [7].

The reproductive history of a woman is closely linked to breast cancer risk [8–10]. The first full-term pregnancy (FTP) is a key event in the determination of the fate of the mammary gland in a woman. Pregnancy exerts a protective effect in women who go through a FTP before the age of 25 [8, 11, 12]. Moreover, multiple FTPs significantly decrease the risk of developing breast cancer even further, whereas postponement of the first FTP to the mid-thirties increases the risk compared to nulliparous women [8, 13]. Pregnancy is a hormonally complex process involving a perfect synchronization of estrogen, progesterone and human Chorionic Gonadotropin (hCG) levels. These hormones are essential for the maintenance of pregnancy and breast development in preparation for milk production [14, 15]. Research shows that primiparous women younger than 25 years of age who have high levels of hCG during the first trimester have a 33% decreased breast cancer incidence in their postmenopausal years [9, 16]. As described by our group and others, completion of pregnancy and subsequent breastfeeding for several months, induce long-lasting molecular changes in the mammary gland [17, 18]. These changes result in a significant reduction in the incidence of all types of breast cancer [19–21]. Notably, long noncoding RNAs (lncRNAs) are genetic regulators of the molecular changes that occur by the physiological events of pregnancy [22, 23]. Noncoding RNAs, transcripts of RNA that do not code for a protein, were once thought of as the “dark matter” of the genome, but it is becoming increasingly clear that they play major roles in gene regulation [24]. These RNA transcripts can be categorized into two groups: micro RNA (18–22 nucleotides in length) and long noncoding RNA (lncRNA; arbitrarily classified as equal or greater than 200 nucleotides in length) [24]. LncRNAs have diverse gene expression regulatory functions including transcriptional regulation, post-transcriptional regulation, or direct regulation of proteins [24]. When these functions go awry, however, many necessary biological functions can be negatively affected, and this can result in disease progression, including oncogenesis and cancer progression. LncRNAs constitute a key layer of genome regulation in diverse biological processes and disease. Chromatin modifiers have been associated with lncRNAs to form a complex which can then target specific genomic regions to modify gene transcription in *Cis* or in *Trans* [25, 26]. The further we understand and study these functions and mechanisms, the closer we can get to understanding how lncRNA can be used to prevent, screen for, or be used as therapeutics for breast cancer [27]. Our RNA sequencing analysis showed that there are 42 differentially expressed lncRNAs between parous and nulliparous women. LncEPCAM/LncE – also known as BC200 -, upregulated in the breast tissue of nulliparous women, was selected for further study using a variety of molecular techniques in human epithelial breast cells to determine its relevance in breast cancer and breast cancer prevention. LncEPCAM spans a 13 kb region which produces 3 transcripts of variable lengths (13 kb, 900 bp and 200 bp). The main expression in our dataset derives from the 200 bp long region within the 13 kb region. Further analysis determined this is a previously discovered but poorly studied 200 nt lncRNA named BC200, also known as BCYRN1. For simplicity, LncEPCAM – abbreviated lncE – will be described by its more common name BC200. There are a few publications reporting BC200 RNA as an oncogene, highly expressed in invasive breast carcinomas [28] and other human tumors [29]. In 2004, Iacoangeli et al. suggested that the presence of BC200 in Ductal Carcinoma In Situ (DCIS) was a prognostic indicator of tumor progression [28]. BC200 has the potential to be a molecular tool in the prevention, screening for, diagnosis and prognosis of breast cancer. Our results show that lncE or BC200 is upregulated in the breasts of nulliparous women, and breast cancer cells and tissue. Overexpression of BC200 produces increased proliferation, migration, and invasion in luminal and triple negative breast cancer. Also, overexpression of BC200 increases tumor growth rate in SCID mice. The downregulation of CALM2, a calcium binding protein responsible for proliferation, apoptosis, and cell cycle development [30], as a consequence of BC200 overexpression, may in part explain the phenotypic changes observed in these breast cancer subtypes. In addition, the physiological role of this gene in the normal breast of nulliparous women may be a contributing factor in the increased susceptibility of these women to breast cancer.

## Methods

### Data and human breast sample collection

Three breast core needle biopsies from 8 parous and 8 nulliparous women were obtained. One core was fixed for histological analysis and the remaining cores were used for subsequent RNA extraction [31]. From this set of samples, RNA samples were used to prepare the libraries and run the RNA sequencing (RNAseq) for this project.

All volunteers who were eligible had signed an informed consent and completed a questionnaire that collected data on reproductive history, medical history, family background of cancer, use of tobacco, use of oral contraceptives (OC), and/or use of hormone replacement therapy (HRT) [31] - (FCCC IRB#02–829).

### Library preparation

Total RNA from the core biopsies was isolated using the Qiagen All prep RNA/DNA Mini Kit according to the manufacturer’s instructions (Qiagen, Alameda, CA). RNA quantity was assessed using NanoDrop v3.3.0 (NanoDrop Technologies, Wilmington, DE) and quality was assessed by means of the Agilent 2100 Bioanalyzer (Agilent Technologies, CA). Only high quality RNA was used for library preparation.

Between 200 ng-1 μg total RNA was used for RNAseq library preparation by following the Illumina TruSeq RNA v1 sample preparation guide. RNAseq libraries were quantified by Qubit (Life Technologies), pooled for cBot amplification and subsequent 50 base paired-end sequencing was performed using Illumina HiSeq 2000 platform.

Accurate quantification of the number of amplifiable molecules in the library was critical to the outcome of sequencing results on Illumina next-generation sequencing platforms. cDNA quantity was determined by q-PCR using SYBR Green I dye. 1:8000 dilutions were done to the library and samples were run in triplicates. The average was used to determine the library’s final concentration.

### RNAseq and RNAseq data analysis

RNAseq data was generated using Illumina HiSeq 2000. After the sequencing run, demultiplexing with CASAVA was employed to generate the fastq file for each sample (reads passing filtering can be used as sequence input for alignment). Reads were aligned to the human genome (UCSC hg19 build) using TopHat software [32]. Expression levels were extracted using HTSeq [33] with RefSeq annotation [34]. After removing genes with 0 sequence read from all samples, a total of 20,863 genes were reported for all 16 samples. Data were then normalized by DESeq normalization method [35] and a small pseudo count 10^− 5^ was added before log-transformation. We removed one outlier data point per gene, per test group (parous and nulliparous) before applying the Limma moderate t-test [36] for differential expression analysis. The outlier data point was determined by the farthest distance to the median expression level of the given gene. Forty-two (42) lncRNAs were differentially expressed between parous and nulliparous samples using p.value <= 0.05 and fold change > = 2. The samples were run in two different batches that showed no statistically significant difference between them. Thus, the results from the two batches were combined.

### Integrative genome viewer (IGV)

The Integrative Genomics Viewer tool was used to visualize the RNAseq data [37, 38]. RNAseq data from our project was uploaded to the software and allowed for viewing quality of RNAseq data (i.e. coverage), expression for the different samples, exact location of the lncRNAs, length, and sequence, among other features using BED files generated on UCSC Table Browser.

### Tissue culture and human breast samples

#### General tissue culture procedures

All cell lines were obtained from the Cell Culture Facility (CCF) at Fox Chase Cancer Center (FCCC). To maintain the collections’ integrity, cell lines were carefully maintained in culture, and stocks of the earliest-passage cells were stored. All cell lines were maintained in a 37 °C, 5% CO_2_ humidified incubator for the duration of the experiments. All cell lines used are well documented in the literature and most of the cell lines have been authenticated by CCF at FCCC (MCF10A, MCF10F, MCF-7, T-47D, MDA-MB-231, and SK-BR-3).

#### Normal and Cancer breast tissue processing

Frozen tissue was obtained from the Biosample Repository Facility at FCCC. Tissues are from biopsies collected during surgery (FCCC IRB#93–031). Although at the time of pathological processing for storage in the Tissue Bank the samples were separated in normal -or- adjacent-to-the tumor and cancer, we re-evaluated the tissue by Hematoxylin & Eosin (H&E) to only use tissue classified as normal which in fact we could corroborate had a normal-histological appearance. Those bonafide normal breast tissues were selected for comparison of gene expression between them and cancer tissue. Each sample stored in the Tissue Bank at FCCC contains an exhaustive report collected on the patients’ clinical history before surgery and the final histopathological report.

Frozen tissues were embedded in OCT (Optimal Cutting Temperature compound) and placed in cryomolds previous to cutting. Only tissues that showed a clear histology (normal and tumor) were used for further analysis.

#### RT-qPCR

Reverse Transcriptase quantitative PCR with TaqMan primer/probe detection was performed and expression levels of selected lncRNAs were determined in triplicate. Each experiment was also run three times. Primer/Probes were designed with Applied Biosystems custom tool and TaqMan reagents were also obtained from Applied Biosystems. As most of our RT-qPCR targets were novel lncRNAs, we used the lncRNA’s sequence as target information for primer/probe design.

#### Lentiviral infections for overexpression of lncRNAs

We generated lentiviral constructs that contained a green fluorescent protein (GFP) tag to be used for the selection of the cells. The lncRNA full length was cloned into the lentiviral vector (p-GFP-Lenti TR30023 8.7 kb; Origene with CMV promoter-GFP reporter and U6promoter-lncRNA-puromycin selection antibiotic). HEK293T cells were co-transfected with a lentiviral vector and packaging plasmids. Then, 24–48 h later media from the transfected HEK293T cells was collected (which contains lentiviral particles), filtered and concentrated. These viral particles were then used to transduce cells of interest (T-47D and MDA-MB231). These cells of interest (T-47D and MDA-MB-231) were co-transfected in 6-well plates with the lncRNA-GFP lentiviral vector and the packaging plasmid using a lipid based transfection reagent (MegaTran, Origene). Infection efficiencies ranged between 20 and 50% depending on the target cell line. Expression changes were considered significant if they showed a two-fold change in expression compared to GFP controls (cells transfected with the lentiviral vector containing GFP only). Control cell lines or “infection control” (baseline cell line exposed to just the packaging plasmids and transfection reagent but no lentiviral vector) were used to determine the threshold when using flow cytometry for selection. Results shown are a result of infected cells left in culture for 2 weeks, maintained in media with puromycin, to obtain stable cell lines.

#### Flow cytometry

Flow cytometry was used to select for cells which expressed a substantial amount of fluorescence. Control cells or “infected control” were used to determine a threshold each time cells were run through flow cytometry. Briefly, cells were resuspended in complete media containing antibiotics (penicillin, 100 U/ml; streptomycin, 100 μg/ml) to avoid possible contamination during flow cytometry. FACS-sorted cells were then grown in a humidified 5% CO2 37 °C incubator until there were enough cells for experiment, keeping puromycin selection. Before phenotypic experiments, a fraction of cells was used to check lncEPCAM/BC200 overexpression.

#### Fluorescence in situ hybridization (FISH)

In situ hybridization with single molecule RNA against candidate lncRNAs was performed by using labeled complementary Stellaris RNA probes on paraformaldehyde-fixed cells [39]. Hybridization signals were then detected by fluorescence microscopy [40]. A mix of multiple 20-mer oligonucleotides, each labeled with a single Quasar® 670 fluorophore was designed using Stellaris web designer software (www.biosearchtech.com/stellaris-probe-designer) and synthesized. Only the lncRNA sequence is needed to synthesize the FISH probes. LncEPCAM probe was composed of 48 probes (20 nts in length) spanning over the lncEPCAM complete RNA sequence length. For MALAT-1 probe (positive control), the Stellaris FISH probe human MALAT-1 with Quasar 670 Dye was ordered. Adherent cells were grown on cover glass and subsequently fixed and permeabilized. Hybridizations were carried out for 16 h at 37 °C in 50 μl hybridization solution (10% dextran sulfate, 10% formamide in 2X SSC). Samples were then washed, DAPI stained, and imaged.

#### TUNEL assay

To evaluate the cell death induced by the lncEPCAM/BC200 overexpression, we analyzed the overexpressing cells using Terminal Deoxyribonucleotide Transferase-Mediated dUTP modified nick-end labeling (Click-iT® Plus TUNEL assay for In Situ Apoptosis Detection, Alexa Fluor® 594 dye). A negative and a positive control (using DNAase to produce DNA fragmentation, Promega, Wisconsin) were simultaneously prepared along with our generated cell lines. Fluorescence microscopy was used to capture the image of the TRITC-labeled TUNEL-positive cells. Imaging specifics: The microscope - Olympus BX53 fluorescent microscope (Olympus); the camera - RetigaTM 2000R Fast 1934 Digital CCD Camera-Monochrome (QIMAGING Corporation, Burnaby, BC, Canada); the software - MetaMorph Software version 7.7.8.0 (Molecular Devices, Sunnyvale CA).

#### MTT assay

Cell proliferation was assessed by measuring tetrazolium MTT (3-(4,5-dimethylthiazolyl-2)-2,5-diphenyltetrazolium bromide) absorbance using Vybrant MTT Cell Proliferation Kit (Molecular Probes, Eugene, OR) [41]. For this purpose the cells were seeded in 100 μL culture medium into costar 96-well flat bottom tissue culture plates at an optimal density per cell line (2000–4000 cells/well) to have a 50–80% confluent culture by the time of measurement [42]. MTT was measured in 3 consecutive days starting the day after seeding to measure effect of overexpression of lncRNA in the cells. Optical density was read at 570 nm using Epoch Microplate Spectrophotometer (Biotek, Winnoski, VT).

#### Proliferation, migration and invasion by real time cell analysis (RTCA)

Cell assays were performed using a Real Time Cell Analysis (RTCA) machine at the CCF at FCCC. The xCELLigence® RTCA DP instrument uses noninvasive electrical impedance monitoring to quantify cell proliferation, and attachment quality in a label-free, real-time manner. Cells overexpressing lncEPCAM/BC200 in a specific cell line were plated in RTCA electronically integrated 16-well plates. RTCA provides data in real time and can be programmed to provide data in various short time regimes. Migration and invasion were evaluated every 15 min; proliferation was evaluated every hour. For invasion assay, the 16-well integrated Boyden chamber (CIM plate) was coated on the upper chamber with matrigel 1:40 (matrigel:serum free media). The lower chamber contains culture media with 10% fetal bovine serum (FBS). The two chambers were assembled and serum starved cells were added to the upper chamber. The gold microelectrodes collect data at specified intervals and real time curves are created by xCELLigence software (aceabio.com/products/xcelligence-rtca).

#### Xenograft study

Female CB17/SCID mice of 6–8 weeks of age were obtained from FCCC animal facility. The tumorigenic ability of the cell lines modified by the overexpression (OE) of the selected lncRNA (lncEPCAM/BC200) was tested in 6–8 week old female CB17/SCID mice. All the animal experiments were carried out at the Laboratory Animal Facility of FCCC, following the protocol approved by the Institutional Animal Care and Use Committee (IACUC #16–05). Cells which overexpressed BC200 were injected subcutaneously in the mammary fat pad of the abdominal region of the mice and tumors were measured three times a week and excised when they reached a maximal diameter of 10 mm [43]. The mice received intraperitoneal injection of 90 mg of Ketamine/Kg of body weight (1:10 Xylazine/Ketamine solution). After collection of tumors in the mammary fat pad, the thoracic cavity was opened followed by pneumothorax puncture for death assurance following the FCCC Guidelines for Euthanasia. At least 5 mice were evaluated in each separate xenograft experiment.

Specifically, we subcutaneously inoculated 2 × 10^6^ lncEPCAM/BC200 OE MDA-MB-231 cells and 5 × 10^6^ lncEPCAM/BC200 OE T-47D in 100 μl of matrigel in the mammary fat pad of CB17/SCID mice [44]. T-47D is an estrogen receptor positive cell line. The growth of these cells depends on higher levels of estrogen than what CB17/SCID mice produce. Thus, for T-47D xenograft models, implantation of a subcutaneous 17-β-estradiol-releasing pellet was required for the formation of tumors [44, 45]. The pellets were prepared in house under sterile conditions for a final concentration of 0.75 mg of estrogen/pellet. Tumor response was evaluated by determining the number of mice which developed a tumor and the size of each tumor. Tumor volume was calculated as follows: 0.5 × L × W2, where L (length) and W (width) are the large and smaller diameters. Tumors were processed for H&E and immunocytochemical studies. All organs (lungs, brain, liver, kidneys, spleen, bladder, uterus & ovaries) were processed for H&E to evaluate tissue abnormalities or metastasis due to tumor formation in the mammary fat pad.

### Statistical analyses

Data were analyzed using the unpaired Student’s t-test. Values represent the mean ± Standard Deviation from one representative experiment of three independent experiments. Tests were performed separately for each cell line. The p.value of 0.05 or less was considered statistically significant. All in vitro experiments were performed at least three times.

For the xenograft studies, using two sample two-sided t-test with a 5% Type I error, with 6 animals in each arm of MDA-MB-231 xenograft studies, we were able to detect differences in tumor size with at least 80% power. With 5 animals in each arm of T-47D xenograft studies, we were able to detect differences in tumor size with at least 90% power.

## Results

### Identification of differentially expressed lncRNAs in the nulliparous breast

**Fig. 1:**
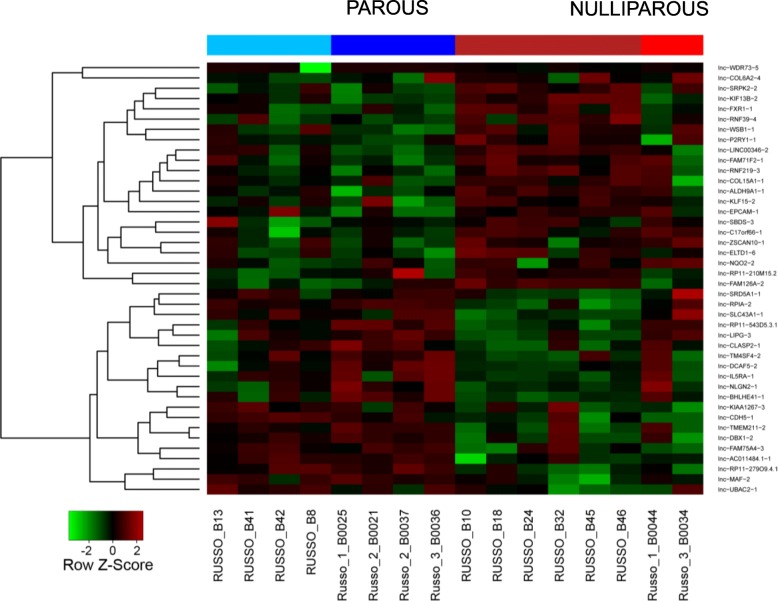
Heatmap of differentially expressed lncRNAs in the breast tissue of parous and nulliparous women. From a total of 42 differentially expressed lncRNA regions, 21 were downregulated in parous breast (in green) and 21 were upregulated (in red). Fold change > = 2.0 & adjusted *p*-value < = 0.05. The two colors under each group (for example, parous = 2 shades of blue) indicate 2 batches sequenced at different times. All other factors were kept them same

By comparing the RNA sequencing (RNAseq) data from 8 parous and 8 nulliparous postmenopausal women, we have determined the significant upregulation and downregulation of a number of long non-coding RNAs (lncRNAs or lnc-RNAs). The RNAseq results of the expression of lncRNAs in parous and nulliparous women are depicted in Fig. 1. We identified 42 differentially expressed lncRNAs (fold change > = 2; adjusted *p*-value <= 0.05) from which 21 were downregulated and 21 were upregulated in parous breast tissues.

### BC200 is upregulated in breast cancer cell lines

Our initial analysis revealed 21 novel lncRNAs which were highly upregulated in nulliparous women. Literature search determined that all 21 lncRNAs were novel transcripts. Thus, we decided to study their relevance in breast cancer by evaluating their expression in breast cancer cells and breast cancer tissue. The chromosomal location for each lncRNA was obtained from LNCipedia (https://lncipedia.org) and the coverage was viewed at an 800 bp resolution. Ideal coverage was defined as regions which showed high levels of readings consistently over a distance of at least 150 bp, preferably with a defined difference in expression between parous and nulliparous samples. After bioinformatics analysis we selected ten lncRNAs to be tested in vitro. These lncRNAs were selected taking into account quality and coverage of RNAseq data in the regions where the lncRNAs’ sequence lie and the ability to generate specific primer probes for RT-qPCR.

**Fig. 2:**
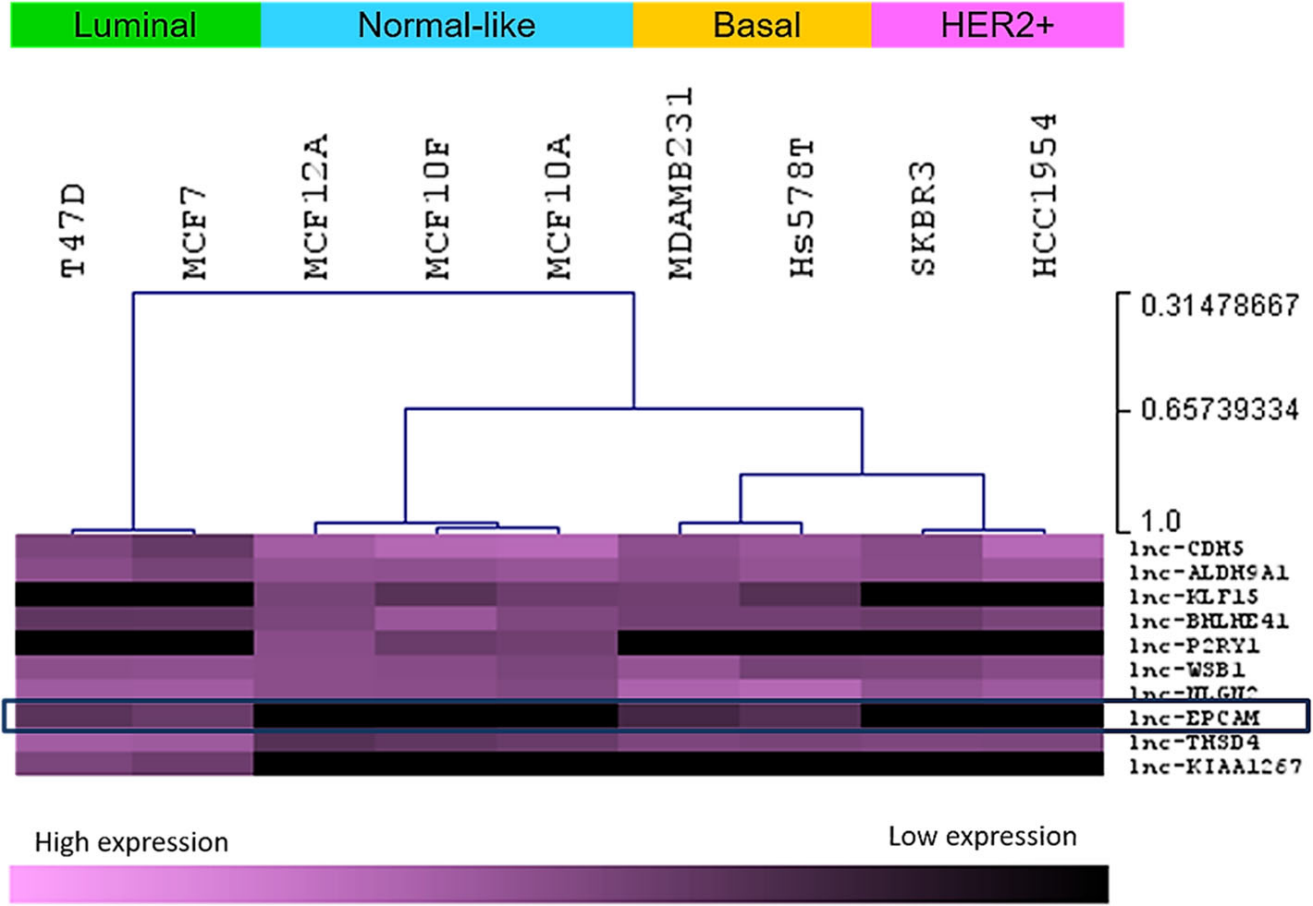
Expression levels of ten lncRNAs in breast cell lines. LncRNA expression is clustered according to breast cancer subtype

The expression of ten lncRNAs were evaluated in commercial and well characterized cell lines that represent different molecular subtypes of breast cancer (Fig. 2). We found that a previously identified but poorly studied lncRNA, called LncEPCAM/BC200, is upregulated in luminal and basal/triple negative breast cancer cells compared to normal immortalized cell lines such as MCF-10A, MCF-10F, and MCF-12A (also described as “normal-like”).

LncEPCAM, located on chromosome 2, spans a 13 kb region and generates 3 transcripts (https://lncipedia.org/db). From our RNAseq results, we determined that the main differential expression in our two sample sets derives from a 200 bp long region within the 13 kb region. As mentioned before, further analysis determined this is a previously identified but poorly studied 200 nt lncRNA named BC200 (Table 1). As annotated in LNCipedia, BC200 is also known as BCYRN1 RNA, BC200a, NCRNA00004, LINC00004; BC200 is transcript 3 of lncEPCAM [46, 47]. Databases have updated its name and is now found in NCBI and lncRNA databases associated with a few publications with the name BC200 or BCYRN1.

**Table 1:** LncEPCAM/BC200 characteristics. Genomic information and RNAseq data. Fold Change (FC) is relative to nulliparous women. A FC < 1 represents a lncRNA downregulated in parous women (i.e. upregulated in nulliparous women), such as BC200

Chromosome	2.p21
Location	chr2:47558199–47,571,656
Length (gene/transcript)	13,458 bp/200 bp
# of Exons	1
Strand	+
Type	intergenic
Transcriptional Direction	Sense to EPCAM
Fold Change	0.48
Regulation	Upregulated in Nulliparous
P-value	0.0041

### BC200 is upregulated in breast cancer tissue

**Fig. 3:**
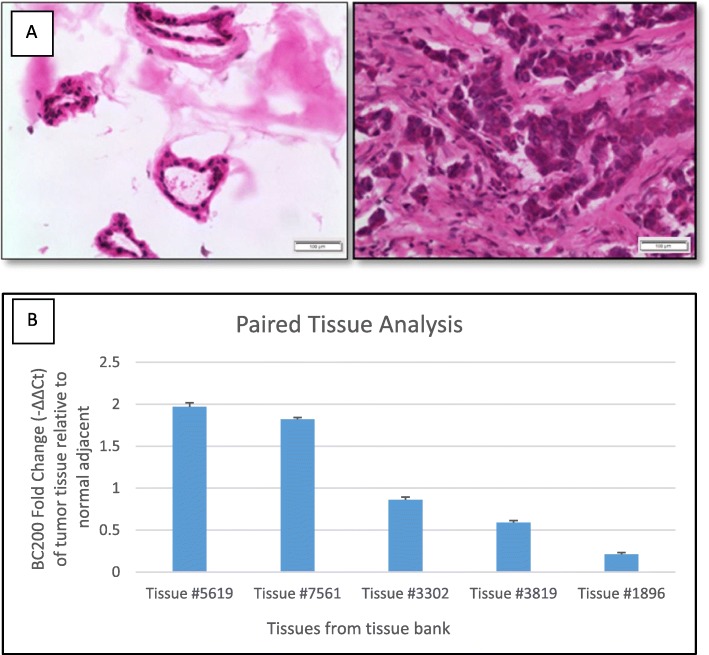
Breast cancer tissue quality evaluation and lncEPCAM/BC200 expression in breast cancer tissues. **a** H&E staining of breast cancer tissues. Expected tissue structures and morphology for normal tissue (left panel - ducts and ductules) and tumor tissue (right panel) (100x magnification). **b** Expression of BC200 in cancer tissue. BC200 is upregulated in 5 out of 10 patients’ breast tumor compared to normal adjacent tissue (BC200 is not expressed in the other 5 tumor tissues). Fold change was determined by the following equations: ΔCt = Ct_gene – Ct18S; ΔΔCt = ΔCt_gene – ΔCt_GFP; Fold change = 2^(−ΔΔCt)^ where 18S was used as housekeeping gene. Error bars indicate standard deviation between three technical replicates

Breast tissue from the Biosample Repository Facility at FCCC was used to determine the level of expression of lncEPCAM transcript 3 (i.e. BC200). The Biosample Repository Facility maintains a record of patients who donate cancer tissue and normal adjacent tissue. Each tissue is collected and stored according to FCCC guidelines and patients’ characteristics are recorded. Only tissue labeled as “normal adjacent” which showed duct and ductule formation (anatomic characteristic of normal breast) were selected for analysis of lncRNA expression. Ten paired cancer-adjacent tissue samples passed our stringent tissue quality control. A representative section of breast tissue from a patient is shown in Fig. 3a. In 5 out of these 10 patients, we observed higher expression of BC200 in the tumor compared to normal adjacent tissue. Further analysis determined ER, PR and HER2 status among a plethora of other characteristics. Thus, we were able to evaluate whether receptor status had a correlation with the lncRNA expression levels in the evaluated patients. We did not find a correlation between BC200 and its receptor status in the 10 breast tissue pairs analyzed. For all three breast cancer subtypes, ER + PR + HER2+, ER + PR + HER2-, and ER-PR-HER2- we found BC200 upregulated in tumor compared to normal adjacent tissue (Fig. 3b**)**.

The increased expression levels of BC200 in breast cancer cell lines and breast tissues suggests that this lncRNA may be implicated in breast cancer. Gene expression regulators like lncRNAs, have been described to influence gene expression even when their expression is slightly increased. The fact that BC200 showed increased expression in half of our samples (and was not expressed in the rest) suggested a potential role as a cancer progression regulator. Therefore, BC200 was further investigated in its relevance to breast cancer and its potential to become a biomarker of prevention. Representative cell lines of common breast cancer subtypes such as MCF-7 (luminal type A), T-47D (luminal type B) and MDA-MB-231 (triple negative) were used to determine the relevance of BC200 in a cellular context.

### BC200 is localized in the nucleus and cytoplasm of ER+ and TNBC cells

**Fig. 4:**
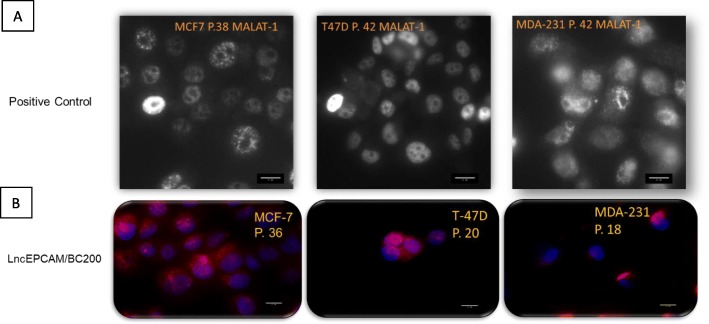
LncRNA expression in cancer cells. **a** MALAT-1 expression in luminal (MCF-7 and T-47D) and triple negative breast cancer (MDA-231: MDA-MB-231) cell lines. MALAT-1 RNA was tested to determine the level of expression of this abundant lncRNA used as positive control. MALAT-1 is a nuclear lncRNA. **b** LncEPCAM/BC200 expression in luminal and triple negative breast cancer cell lines. LncEPCAM/BC200 is both nuclear and cytoplasmic. All images were taken at 400x magnification

RNA in situ hybridization was used to determine BC200’s cell localization. Determining a lncRNA’s localization in the cell, is an indicator of potential function. Low abundance RNAs, such as BC200, are hard to detect unless sensitive methods are used to amplify the signal, without compromising specificity. The careful design of Stellaris specific probes led to the identification of BC200’s localization in cancer cells as shown in Fig. 4. LncRNA MALAT-1 was used as positive control for these reactions as it is abundantly expressed in most cancer cell lines [48]. We confirmed that MCF10A does not express BC200 (data not shown) which goes along with the results obtained by RT-qPCR. BC200 is both nuclear and cytoplasmic in cancer cell lines.

### BC200 overexpression increases cell survival and proliferation

To evaluate if lncE/BC200 has an effect on the phenotype of the cancer cell, we performed phenotypic assays after manipulating its expression. A scrambled negative control (Inf Ctrol or infection control) and a GFP empty vector were added to each experiment to determine the effects of infection and introduction of a 8.0 kb plasmid in the cells. The cells were harvested after infection and the overexpression efficiency was determined via quantitative real-time PCR before using the cells for phenotypic assays. Expression changes were considered significant if they showed at least a two-fold increase in BC200 expression compared to the GFP-empty vector.

**Fig. 5:**
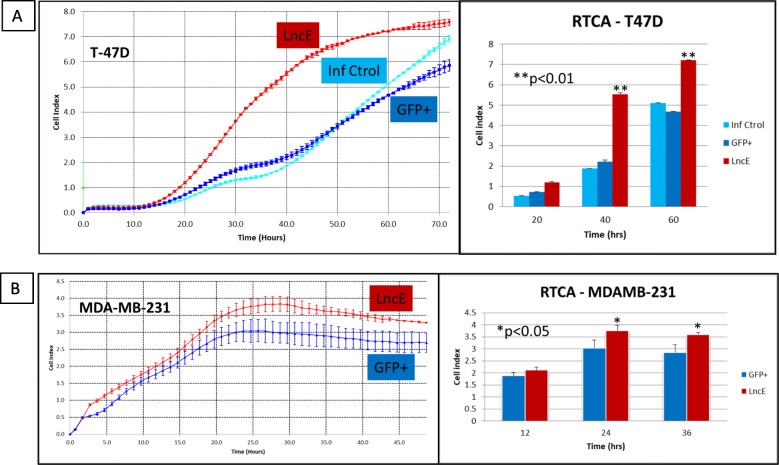
Proliferation of T-47D and MDA-MB-231 cells. **a** Proliferation rate of T-47D by RTCA. Twenty thousand (20,000) cells/well were plated and followed for 72 consecutive hours with data collected every hour; 4 replicates per construct. **b** Proliferation rate of MDA-MB-231 by RTCA. Fifteen thousand (15,000) cells/well were plated and followed for 48 consecutive hours with data collected every hour; 4 replicates per construct. Cells were recorded for at least 48 h – depending on proliferation rate - to determine proliferation rates of cells overexpressing different constructs (Inf Ctrol: no construct or scrambled; GFP+: GFP-expressing vector/empty vector; LncE: lncEPCAM/BC200 overexpressing cells). Left panel is the graph obtained in real time. Right panel represents results from the left panel at specified time points. Results are representative of 3 independent infections (*n* = 3). *p.value (p) < 0.05; **p.value (p) < 0.01 (Inf Ctrol for MDA-MB-231 curve overlapped with MDA-GFP+ and was removed from graph for clarity)

Proliferation was measured by two methods as described in Materials and Methods section. Figure 5 shows proliferation rates of T-47D (Fig. 5a) and MDA-MB-231 cells (Fig. 5b) infected with BC200 construct. Proliferation measured using MTT method at 24 h, 48 h and 72 h post-plating showed similar results (data not shown) compared to real time cell analysis (RTCA). Repeat experiments (infections #1, #2 and #3) gave similar results with exponential growth starting at 20 h and all cells converging at cell index 7 – approximately 1.5 × 10^5^ cells - after 72 h of incubation). BC200 promotes proliferation in both luminal (T-47D) and TNBC (MDA-MB-231) cells as determined by MTT and RTCA methods.

### BC200 overexpression increases cell migration and invasion

**Fig. 6:**
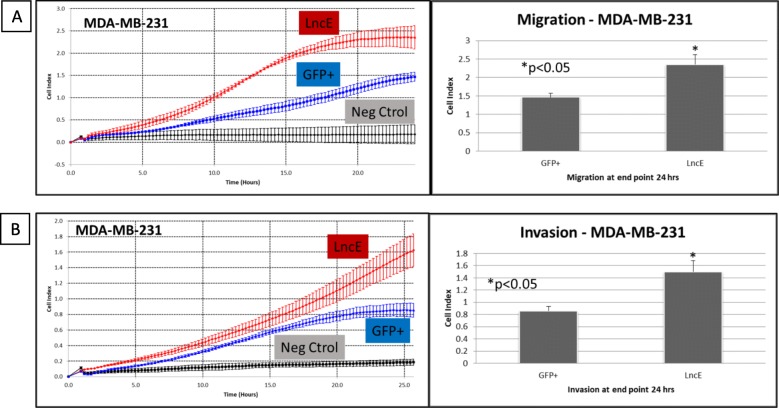
Effect of BC200 on (**a**) migration and (**b**) invasion. MDA-MB-231 cells overexpressing BC200 were subjected to real time cell analysis migration (upper left) and invasion (lower left). Left panels (A and B) show the real time results of cells being recorded every 15 min for 24 h. Right panels (A and B) show results at end point (24 h after seeding 20,000 cells on wells for migration – or wells coated with matrigel for invasion). Results are representative of 3 independent cell infections (n = 3) with average of 4 replicates in each independent experiment. LncE = lncEPCAM = BC200; Neg ctrol = negative control – no serum added to the lower chamber of the RTCA plates. For the invasion experiment, twenty thousand (20,000) cells/well were seeded on matrigel coated wells and were let to invade through the upper chamber to the lower chamber for 24 h

The xCELLigence RTCA instrument from Roche Applied Science was used to determine how lncE/BC200 affects migration and invasion of MDA-MB-231, cells that are considered highly aggressive. We confirmed that MDA-MB-231 baseline cells (and MDA-MB-231 Inf Ctrol) migrate and invade at a similar rate as MDA-MB-231 containing the GFP marker. MDA-MB-231 cell line is widely reported as highly migratory and invasive [49–51] due to the release of ample levels of MMP-9 [52] and other membrane matrix metalloproteinases [53]. Figure 6a shows how the migration rate of MDA-MB-231 is affected by the presence of BC200 and Fig. 6b shows how invasion is similarly affected. More cells migrated and invaded in MDA-MB-231-lncE compared to MDA-MB-231-GFP. The high expressing E-cadherin cell line T-47D has very little to no migratory and no invasive capacity [54–56] unless transformed with KRas or NRas [57]. They are considered non tumorigenic (tumors take more than 10 months to grow in nude mice) unless supplemented with exogenous estrogen [45]. We tested if the introduction of BC200 modified its non-migratory and non-invasive characteristics. T-47D cells infected with BC200 showed the same low migratory and low invasive effect as T47D-GFP+ and the negative control (with serum free media in both upper and bottom chamber, and T-47D cells were plated in the upper chamber) followed by the RTCA system in a 48 h period. After 48 h serum deprived T-47D cells start dying. We concluded that the presence of BC200 did not modify non-migratory and non-invasive capacity in the T-47D cell line.

### BC200 may regulate *in Cis* suppressing apoptosis in ER+ and TNBC cells

**Fig. 7:**
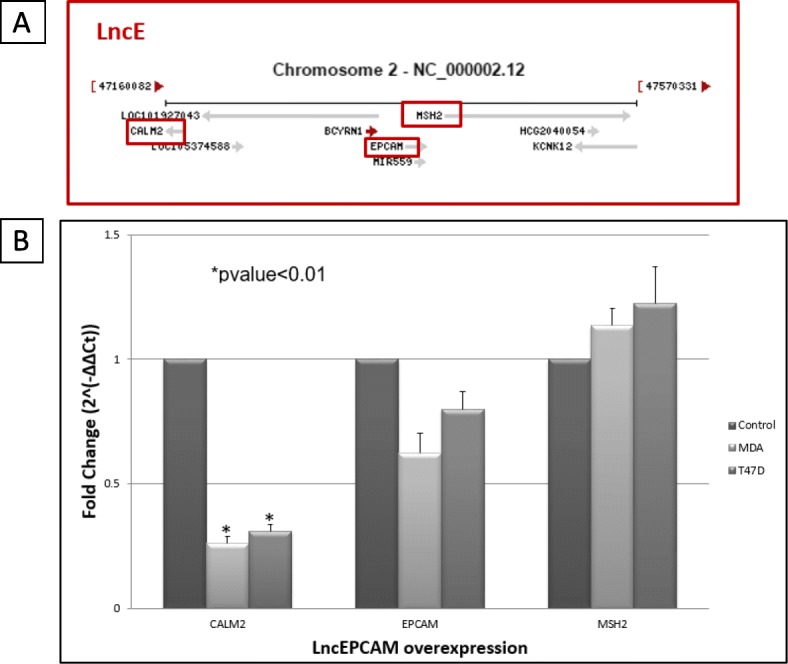
LncEPCAM locus. **a** Genomic region around lncEPCAM. NCBI representation of lncEPCAM/BC200/BCYRN1 genomic neighborhood. CALM2, EPCAM and MSH2 were selected to be further evaluated. **b** Evaluating *Cis* regulation. Effect of lncEPCAM/BC200 overexpression on nearby genes in MDA-MB-231 (MDA) and T47D cell lines. Fold change was determined by the following equations: ΔCt = Ct_gene – Ct18S; ΔΔCt = ΔCt_gene – ΔCt_GFP; Fold change = 2^(−ΔΔCt)^ where 18S was used as housekeeping gene and Ct_GFP corresponds to threshold of the gene in cells that express GFP. Error bars indicate standard deviation between two independent experiments. MDA: MDA-MB-231; T-47D: T-47D

The expression of three genes located near BC200 were examined to determine if it was plausible that BC200 was regulating them in *cis* manner. The genes are EPCAM, CALM2, and MSH2 (Fig. 7). Using IGV to study our parous vs. nulliparous sequencing dataset and combining IGV analysis with RNAseq data, we determined that EPCAM was 36.68% (pvalue = 8.35*10^− 15^) more expressed in parous women. CALM2 leaned towards a nulliparous favored expression of 58.98% (pvalue = 5.18*10^− 4^). Finally, MSH2 is 54.42% more expressed in parous women (pvalue = 0.0011).

CALM2 is a calmodulin, a calcium binding protein responsible for cell signaling, proliferation, apoptosis, and cell cycle development [30]. In breast cancer cells, CALM2 directly binds to death receptor-5 (DR5) in a calcium dependent manner leading to the formation of death inducing signaling complex for apoptotic signaling [58]. When BC200 is overexpressed, CALM2’s expression decreased more than half in MDA-MB-231 and T-47D compared to respective control (Fig. 7b). This preliminary data could hint on how an increased expression of BC200 in nulliparous women or cancer cells may be *Cis* regulating the expression of CALM2. Increased levels of CALM2 have been linked to a regulation of cell apoptosis in breast cancer cells.

EPCAM or Epithelia Cell Adhesion Molecule is a type I transmembrane protein that is expressed in the majority of normal epithelial tissues and is overexpressed in most epithelial cancers including breast cancer [59, 60]. However, EPCAM’s expression levels do not significantly change when BC200 is overexpressed in MDA-MB-231 and T-47D.

MSH2 is a homolog of the *E. coli* mismatch repair gene mutS. Heterozygous germline mutations in any of the mismatch repair (MMR) genes - MLH1, MSH2, and MSH6 - cause Lynch syndrome, an autosomal dominant cancer predisposition syndrome conferring a high risk of colorectal, prostate and endometrial cancers, among others [61, 62]. MSH2’s expression levels do not significantly change when BC200 is overexpressed in MDA-MB-231 and T-47D.

### BC200 overexpression enhances tumor growth in xenograft mouse model

A viable single-cell suspension of T-47D or MDA-MB-231 cells overexpressing BC200 (T-47D-lncBC200 and MDA-MB-231-lncBC200) in 100 μL of PBS was mixed with 100 μL matrigel. Cells were then injected into the mouse mammary fat pad. No major changes were observed in the weight of mice for MDA-MB-231 xenograft experiment. The average weight was about 20 g ± 3 g and all animals looked healthy at the time of sacrifice.

**Fig. 8:**
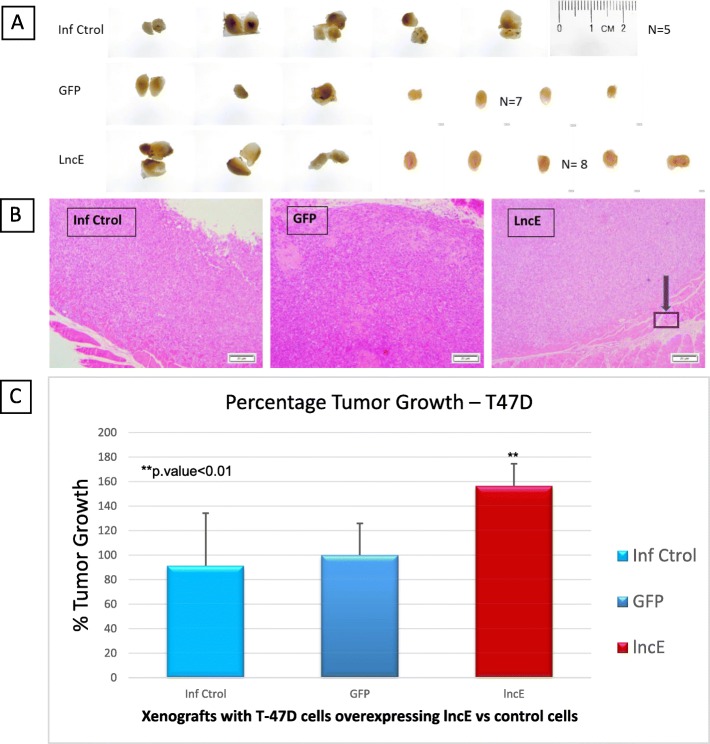
Mice T-47D tumors overexpressing lncE/BC200 and histological sectioning. **a** Tumors dissected from each mice at 4 weeks. **b** Representative H&E stained section of poorly differentiated tumor at end point (4 weeks) (40x magnification). The tumor has invaded to the muscle (squared section). **c** Percent tumor growth at end point. **p.value < 0.01. lncE: lncEPCAM. **a** shows the dissected tumors at end point for T-47D. H&E staining of poorly differentiated adenocarcinoma is shown in **b**. Results in **c** are expressed as percentage tumor growth as two separate experiments’ results were combined to increase the power. Mice containing T-47D-lncEPCAM cells in the mammary fat pad (**c**), grow significantly larger tumors compared to T-47D-GFP in the 4-week period of the experiment

Xenografts experiments using T-47D cell line require the extra step of estrogen pellet insertion as cells do not grow (or grow very slowly) without estrogen stimulation. This requires survival surgery a couple of days before cell injection and as a consequence more handling and potential exposure to immunocompromised animals. Survival surgery went smoothly and mice looked healthy and healed well after surgery. However, 2 weeks after surgery (1 week after clip removal) a few mice started to lose weight. Mice were monitored and eventually 4 mice had to be sacrificed due to extreme weight loss. We also noticed drier skin and rough fur in these animals. These events may all be a consequence of estrogen exposure. Thus, as all these animals were either in the GFP-control group or the BC200 group, we decided to repeat these 2 groups with 5 mice/group for a total of 7 mice in the GFP-control group and 8 mice in the BC200 group. In summary, T-47D experiment was repeated with 5 more animals (and results were combined leading to % Tumor Growth - Fig. 8) because severe weight loss was observed due to higher levels of estrogen in the body as a result of the presence of estrogen pellet.

Combining both rounds, we observed that BC200 overexpression in T-47D cell induced over 50% increase in tumor growth (p.value< 0.01) in the 4-week period of the experiment (Fig. 8). Additionally, we observed that T-47D cells which overexpress BC200 have invaded the muscle (purple arrow in Fig. 8b), suggesting that BC200 increases the invasiveness of T-47D cells in vivo.

**Fig. 9:**
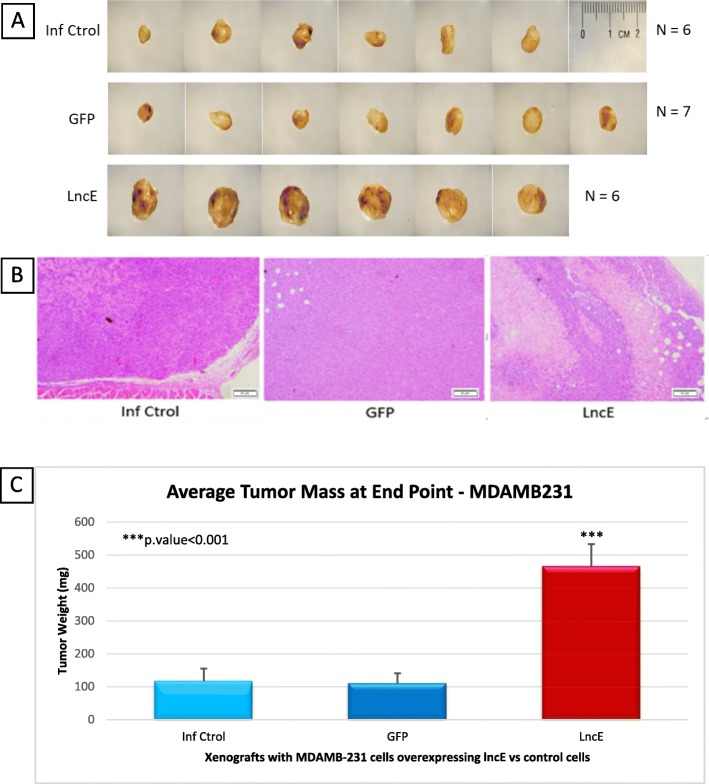
Mice MDA-MB-231 tumors overexpressing lncE/BC200, and histological sectioning. **a** Tumors dissected from each mice at end of 4 weeks. **b** Representative H&E stained section of poorly differentiated tumor at end point (4 weeks) (40x magnification). **c** Tumor weight at end point. ***p.value < 0.001. lncE: lncEPCAM. **a** shows the dissected tumors at end point for MDA-MB-231. H&E staining of poorly differentiated adenocarcinoma is shown in **b**. As **c** shows, mice containing MDA-MB-231-lncEPCAM cells in the mammary fat pad, grow significantly larger tumors compared to MDA-MB-231-GFP in the 4-week period of the experiment

Mice containing xenografts with MDA-MB-231-BC200 cells in the mammary fat pad, grow tumors almost 4.5 times larger than the animals that received MDA-MB-231-GFP in the 4-week period of the experiment (Fig. 9).

In short, we observed that the overexpression of BC200 in both cell lines promotes xenograft growth in CB17/SCID mice.

## Discussion

By comparing the RNA sequencing (RNAseq) data from 8 parous and 8 nulliparous postmenopausal women, we have determined the significant upregulation and downregulation of a number of long non-coding RNAs. We have previously reported significant differences in gene transcription between the postmenopausal nulliparous and parous breast by microarray and qRT-PCR [22, 31, 63, 64]. Otherwise, these two populations are comparable, with similar genetic and geographic background [31]. From our preliminary screening, we found that BC200 was a candidate with tumorigenic characteristics and evaluated it further.

RNAseq identified 42 novel long non-coding regions that were significantly and differentially expressed between parous and nulliparous breast tissue samples [23, 65]. The power of this model is that the lncRNAs were discovered directly from a cohort of 16 women who volunteered for breast biopsies of healthy tissue. To the best of our knowledge, this is the first time that normal tissue in two different physiological conditions (pregnancy vs. no pregnancy) has been studied to identify noncoding regions that are differentially expressed between the two groups. Although this is a small cohort, these findings highlight the differences among apparently similar tissue (from the histological point of view). Given the plethora of potential functional roles lncRNAs have, we believe the lncRNAs identified in this study are a class of genetic regulators that is largely untapped. In the present work, for the first time, we report the differences observed in the differential expression of lncRNAs in these two groups of women and thus increase our understanding of the molecular and epigenetic processes that may lead to breast cancer prevention in parous women. In the context of healthy tissue, these lncRNAs may be highlighting the predisposition nulliparous women have over the increased risk of developing breast cancer in their postmenopausal years. In the context of disease, these lncRNAs may serve as drivers of cancer as well as potential therapeutic entry points. In order to study these lncRNAs in the laboratory, we turned to a normal vs. cancer setting to evaluate their expression levels, relevance and potential applicability of the information discovered in a parous vs. nulliparous setting.

When analyzing the characteristics and location in the genome of these 42 lncRNAs, we discovered that lncEPCAM (also known as BC200) had only been reported in a handful of papers. Its potential implication in breast cancer had been reported a few years back [29]. However, this lncRNA had mainly been implicated in brain pathology such as in Alzheimer’s disease [66]. Recently, it has become clear of the relevance of BC200 as a key regulator in cancer [67–69], specifically breast cancer [70–72]. However, the findings are still in its infancy.

Our in vitro data show that BC200 is not only differentially expressed between normal and cancer cells but also cluster the different breast cancer subtypes in luminal, basal/TNBC and HER2+. After successful overexpression of this lncRNA in the selected cell lines, we tested transformation phenotypes.

The luminal cell lines were chosen based on the fact that over 70% of breast cancers are of the luminal type [5]. Triple negative breast cancers (TNBC) accounts for 10–20% of breast cancers and has been found to be associated with younger age, more advanced stage of diagnosis, and no current local treatment except for mastectomy with or without radiotherapy, due to lack of drug-targetable receptors [73]. Although TNBC is sensitive to chemotherapy, survival after metastatic relapse is short, treatments are few, and the response rate is poor and lack durability [73].

We hypothesized that this lncRNA was a key driver in the process of molecular remodeling that occurs in the mammary gland during pregnancy, providing protection against the development of breast cancer. To understand its role in cancer progression, we evaluated the functional consequences of overexpressing BC200 in breast cancer cell lines, both in vitro and in vivo.

Our data show that BC200 is indeed expressed in breast cancer cells. This coincides with the scarce literature reporting BC200 (also known as BCYRN1) expressed in cancer tissue [29]. Importantly, overexpression of BC200 leads to increased proliferation in luminal and basal/TNBC cells. BC200 overexpressing cells show statistically significant increase in migration and invasion in both luminal and TNBC cells. In vivo, BC200 overexpressing cells produce large tumors in the mammary fat pad that invade the abdominal muscle showing the aggressiveness of these cells. Also, our preliminary data in mouse tissue indicate that there are more Ki67 positive cells in MDA-MB-231-lncE and T47D-lncE tumor cells in xenografts than in MDA-MB-231-GFP and T47D-GFP tumors, respectively (data not shown). Although a few publications have described this lncRNA as an oncogene, reporting that BC200 RNA is highly expressed in invasive breast carcinomas [28] and other human tumors [29], it was only recently that a possible mechanism of action for BC200 contributing to breast carcinogenesis was reported [74]. In 2004, Iacoangeli et al. suggested that the presence of BC200 in Ductal Carcinoma In Situ (DCIS) was a prognostic indicator of tumor progression. BC200 had the potential to be a molecular tool in the diagnosis and prognosis of breast cancer [28]. In 2015, a patent by Tiedge et al. suggested BC200 RNA as the diagnostic molecular tool for breast cancer after extracting and measuring the levels of BC200 RNA in whole blood. The authors determined that patients having circulating blood levels of 25x BC200 RNA, compared to control patients with no disease, have an increased risk for the development of breast cancer [75]. This parameter is proposed as an early diagnostic tool, using a sample which is ease to obtain with no or few side effects. Notably this patent is still pending. More recently, Singh et al. published a paper further providing evidence of the role of BC200 in breast cancer. They demonstrated that BC200 contains sequence complementarity to Bcl-x mRNA and thus may facilitate the regulation of alternative splicing of Bcl-x mRNA in ER+ breast cancer cells. The authors also demonstrated that BC200 knockout (KO) suppressed ER+ tumor growth in vivo [74]. Singh et al. determined that BC200 was expressed in MDA-MB-231 cell line but did not follow up as they determined that the expression of this lncRNA in MDA-MB-231 cells was lower than in luminal cells such as MCF-7 and T-47D. In addition to confirming results published by Singh et al. on MCF-7 cells, we expanded the study to T-47D and we determined that similar traits are observed in the TNBC model MDA-MB-231. Thus, the Singh et al. publication served as a solid platform to establish the high relevance of BC200 in breast cancer pathogenesis [74]. They tackled how, mechanistically, BC200 is critical to cell proliferation and survival. By using CRISPR/Cas9 system they knocked out BC200 in MCF-7 cells and showed that the latter produced an increase in the level of pro-apoptotic Bcl isoforms [74]. Although these findings are very enlightening, we demonstrated here that the effect on breast cancer pathogenesis is not only on ER+ breast cancer, but also in TNBC. We believe BC200 effect on breast pathogenesis may not only be limited to regulation of alternative splicing of Bcl-x by BC200 but there are sure other mechanisms contributing to this.

Since the lncRNA field emerged, experts have discussed the importance of findings for genes that are expressed at a low level. It has been proven time and again that tight regulation occurs with genes expressed at low levels, more so in the lncRNA field [76]. BC200 effect on breast pathogenesis may not only be limited to regulation of alternative splicing of Bcl-x by BC200 but there are sure other mechanisms contributing to this. Even with a small sample size for RNAseq data analysis, our cell based model shows that BC200 effect on breast pathogenesis is not limited to ER+ breast cancer. Our data demonstrates that BC200 is highly relevant in TNBC as well. Our preliminary results on *Cis* regulation by BC200 build upon other authors’ findings unmasking the mechanistic regulation of this 200 bp lncRNA. However, further research on BC200’s mechanism of action is needed to confirm these preliminary results.

CALM2, a gene responsible for apoptosis, proliferation, and cell cycle progression [30, 77, 78], is downregulated in both cell lines (T-47D-lncE and MDA-MB-231-lncE) indicating that BC200 may suppress CALM2 expression to deregulate cell cycle progression and apoptosis. In breast cancer cells, CALM2 directly binds to death receptor-5 (DR5) in a calcium dependent manner leading to the formation of death inducing complex for apoptotic signaling [58]. Haddad et al. have suggested that CALM2 is involved in the etiology of breast cancer, especially in African American women, by performing gene-based and single-SNP analyses [79]. CALM2 was included in the study because of calmodulin’s involvement in gonadotropin-releasing hormone (GnRH) signaling. As previously described by Melamed et al., GnRH induces calcium influx, which activates calmodulin, leading to gonadotropin gene expression [80]. Thus, CALM2 may impact breast cancer susceptibility through its effects on hormone synthesis [79]. The observation that CALM2 is downregulated as a consequence of overexpression of BC200 indicates that cells tend to shut down a gene responsible for cell death and controlled proliferation and cell cycle progression in favor of deregulated apoptosis and uncontrolled proliferation, and cell cycle progression. Our results add key pieces to the body of work demonstrating that BC200 plays a critical role in cell cycle progression [81]. The authors also report on the fact that BC200 inhibition is toxic to actively proliferating cells supporting the rationale of targeting this lncRNA in the treatment of not only breast cancer but also a broad spectrum of tumor types where BC200 is upregulated [81].

## Conclusion

Altogether, the overexpression of lncE/BC200 in breast cells shows that this nearly novel lncRNA has a role not only in the development of the neoplastic process but also in how its low-to-insignificant expression in parous women may be causing the protection of breast cancer development during postmenopausal years. Also, here, we have confirmed the relevance of BC200 in luminal breast cancer and for the first time reported the relevance in TNBC. Prospective studies using reported methods to detect the levels of BC200 in blood [75], would confirm its potential as a biomarker in the prognosis of breast cancer development/progression in high risk populations, such as women with a family history of breast cancer and BRCA-1 and/or BRCA-2 mutation carriers. Women with a higher risk of developing breast cancer, such as nulliparous women, may also benefit from this potential biomarker.

## Data Availability

The P/NP datasets used and analyzed during the first portion of this study are available from the corresponding author at FCCC (JR) on reasonable request.

## Abbreviations

BC200: Brain cytoplasmic 200 long non coding RNA
Bcl: B-cell lymphoma
BCYRN1: Brain cytoplasmic RNA 1
BRCA: Breast cancer susceptibility gene
CALM2: Calmodulin
CCF: Cell culture facility
DR5: Death receptor5
ER: Estrogen receptor
FACS: Fluorescent activated cell sorting
FCCC: Fox chase cancer center
FTP: Full term pregnancy
GFP: Green fluorescent protein
GnRH: Gonadotropin-releasing hormone
H&E: Hematoxylin & eosin
hCG: Human chorionic gonadotropin
HER2: Human epidermal growth factor receptor 2
KRas: Kirsten Rat Sarcoma
lncEPCAM: Long non-coding epithelial cellular adhesion molecule
lncRNAs: Long non-coding RNAs
MSH2: MutS Homolog 2
Nras: Neuroblastoma Rat Sarcoma Homolog
OC: Oral contraceptive
OCT: Optimal cutting temperature
PCR: Polymerase chain reaction
PR: Progesterone receptor
RNAseq: RNA sequencing
RTCA: Real-time cell analyzer
TNBC: Triple negative breast cancer
